# Furin-Triggered Peptide Self-Assembly Activates Coumarin Excimer Fluorescence for Precision Live-Cell Imaging

**DOI:** 10.3390/molecules30112465

**Published:** 2025-06-04

**Authors:** Peiyao Chen, Liling Meng, Yuting Wang, Xiaoya Yan, Meiqin Li, Yun Deng, Yao Sun

**Affiliations:** 1 Key Laboratory of Fermentation Engineering (Ministry of Education), National “111” Center for Cellular Regulation and Molecular Pharmaceutics, Hubei Key Laboratory of Industrial Microbiology, School of Life and Health Sciences, Hubei University of Technology, Wuhan 430068, China; chenpeiyao@hbut.edu.cn (P.C.); 102200626@hbut.edu.cn (L.M.); yutingwang0412@163.com (Y.W.); yanxiao4422@163.com (X.Y.); 2 State Key Laboratory of Green Pesticide, International Joint Research Center for Intelligent Biosensor Technology and Health, College of Chemistry, Central China Normal University, Wuhan 430079, China; 18235519526@163.com; 3 Key Laboratory of Flexible Optoelectronic Materials and Technology, Ministry of Education, Jianghan University, Wuhan 430056, China; ustcdy@jhun.edu.cn

**Keywords:** peptide self-assembly, excimer, furin, fluorescence imaging

## Abstract

Monomer-to-excimer transition has become a valuable technique in fluorescence imaging because of its ability to enhance imaging contrast. However, from a practical perspective, the accuracy of excimer formation at target sites warrants further exploration. Enzyme-triggered peptide self-assembly provides a promising solution to this limitation. As a proof-of-concept, in this study, we developed a furin-triggered peptide self-assembling fluorescent probe RF-Cou by coupling a coumarin dye 7-(diethylamino)-2-oxo-2H-chromene-3-carboxylic acid (Cou) with a furin-responsive peptide scaffold for precision live-cell imaging. Upon entering furin-overexpressing 4T1 tumor cells, RF-Cou underwent enzymatic cleavage, releasing an amphiphilic peptide motif and self-assembling into nanoparticles largely concentrated in the Golgi apparatus to confine the diffusion of Cou. During this process, the Cou excimers were formed and induced a red shift in the fluorescence emission, validating the feasibility of RF-Cou in efficient excimer imaging of furin-overexpressing tumor cells. We expect that our findings will highlight the potential of stimuli-responsive small molecular peptide probes to advance excimer-based imaging platforms, particularly for enzyme-specific cell imaging and therapeutic monitoring.

## 1. Introduction

Fluorescence imaging, an advanced in vivo real-time molecular tracking technology, has emerged as a powerful tool in both basic research and clinical practice [1,2]. With its high sensitivity, outstanding spatiotemporal resolution, and non-invasive nature [3], this technique enables dynamic monitoring and diagnostics of tumors at the molecular level, effectively complementing conventional imaging modalities like magnetic resonance imaging (MRI) [4], positron emission tomography (PET) [5], and computed tomography (CT) [6,7]. The rational design of probes represents a pivotal factor governing the efficacy and specificity of fluorescence-based imaging methodologies. However, conventional “always-on” fluorescent imaging probes require prolonged waiting periods for target-site accumulation and systemic clearance of non-targeted probes via metabolism to achieve a sufficient signal-to-noise ratio (SNR) [8]. Therefore, these probes may face significant limitations in practical applications, such as extended imaging time windows, high background noise, and false-positive results [9]. To address these limitations and improve disease-specific imaging performance, increasing research efforts have been directed toward developing activatable fluorescent probes in recent years [10,11]. Scientists have explored various strategies for the development of activatable fluorescent probes, among which, the monomer–excimer transitions have garnered significant attention due to their ability to achieve greater Stokes shifts and longer fluorescence lifetimes compared to the original monomer molecules [12]. There are numerous substances capable of forming excimers, such as pyrene, perylene, and coumarin [13]. Notably, coumarin monomers can form excimers within aggregates, resulting in a red shift in the emission wavelength, which effectively suppresses intrinsic autofluorescence in biological systems, enhances imaging contrast, and improves SNR [14,15]. Activatable fluorescent probes possess inherent responsiveness, with fluorescence signal changes occurring only in response to specific biological alterations within living systems [16]. These microenvironmental alterations, such as dysregulated pH [17], perturbed redox homeostasis [18], abnormally expressed enzymes [19], etc., serve as diagnostically relevant biochemical hallmarks that are intrinsically linked to the underlying pathogenesis at disease sites. For instance, the *trans*-Golgi protease furin is one of the enzymes with obvious characteristics in the proprotein convertase (PCs) family and is involved in the activation of various precursor proteins [20,21]. Its expression is markedly upregulated in multiple solid tumors, including ovarian, breast, and non-small cell lung cancers, where elevated furin levels correlate positively with tumor malignancy grade and metastatic potential [22,23]. Consequently, the development of furin-responsive fluorescent probes holds significant potential for advancing cancer diagnosis, real-time therapy monitoring, and mechanistic studies of pathological progression [24].

Peptides, composed of multiple amino acids linked by peptide bonds, are extensively used in the construction of stimulus-activated fluorescent probes, which typically exhibit good biological activity and excellent biocompatibility [25]. Owing to the designable sequences and modifiable side chains of peptides [26], multifunctional molecules can be constructed through precise amino acid sequence control or side-chain functionalization, providing a molecular foundation for the development of peptide-based enzyme-activated fluorescent materials [27]. Furthermore, peptides with specific sequences possess the ability to self-assemble into supramolecular nanostructures with precisely controlled sizes, shapes, and architectures [28]. The artful integration of a fluorescent reporter, an enzyme-cleavable substrate, and a self-assembling peptide sequence enables the fabrication of an enzyme-triggered peptide self-assembly fluorescent probe. This design facilitates a cascade response of “enzyme recognition—self-assembly activation—signal change”, allowing for precision enzyme monitoring [29]. During this process, the multifunctional peptide selectively accumulates and undergoes in situ self-assembly into nanostructures upon recognition and activation of the disease-specific overexpressed enzyme [30]. The fluorescence properties of the incorporated fluorophores may be modulated upon nanostructure formation, resulting in emission wavelength red-shifting or fluorescence switching (on/off) in response to enzymatic activity. Consequently, it significantly enhances imaging performance at the target site [31]. For example, Yang and collaborators reported a probe [32] that self-assembles into nanofibers after being activated by alkaline phosphatase (ALP), triggering the monomer to excimer transformation and activating the fluorescence of the coumarin excimer with red-shifted emission for tumor imaging. This strategy integrates bio-recognition with imaging signal activation, enabling the development of smart molecular probes for high-contrast disease diagnostics and facilitating spatiotemporally resolved imaging of pathological enzymes [33].

**Figure 1:**
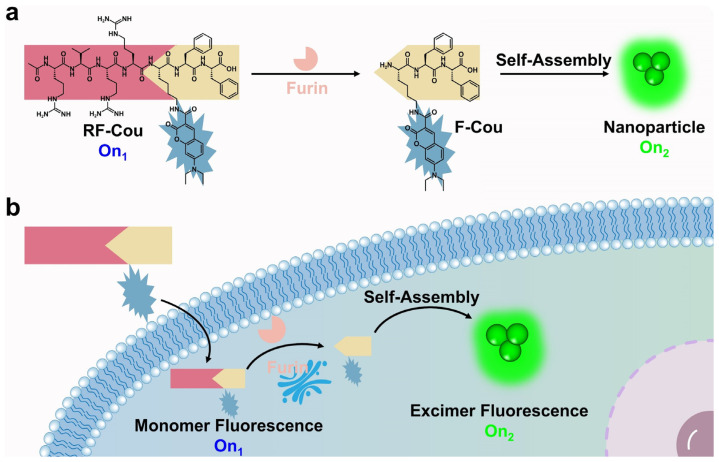
(**a**) Mechanism illustration of furin-triggered excimer fluorescence activation of RF-Cou. Under the specific hydrolysis of furin, RF-Cou generated an amphiphilic structure F-Cou, which further self-assembles into nanoparticles, resulting in the activation of excimer fluorescence. (**b**) Schematic diagram of furin-triggered peptide self-assembling fluorescent probe RF-Cou activates excimer fluorescence for furin imaging in tumor cells.

In this study, we developed a furin-triggered peptide self-assembling fluorescent probe Ac-Arg-Val-Arg-Arg-Lys(Cou)-Phe-Phe-OH (RF-Cou) by conjugating a coumarin dye 7-(diethylamino)-2-oxo-2H-chromene-3-carboxylic acid (Cou) to a furin-responsive peptide scaffold Ac-Arg-Val-Arg-Arg-Lys-Phe-Phe-OH. Upon activation by the furin, RF-Cou generated an amphiphilic structure Lys(Cou)-Phe-Phe-OH (F-Cou), which further self-assembled to form nanoparticles (Figure 1). During this process, the Cou excimer formed, inducing a fluorescence emission red shift from 475 nm (On_1_, monomer) to 550 nm (On_2_, excimer). Using this strategy, we successfully achieved fluorescent imaging of furin-overexpressing 4T1 breast cancer cells, highlighting the potential of peptide-based small molecular probes in advanced biomedical applications.

## 2. Results and Discussion

**Scheme 1:**
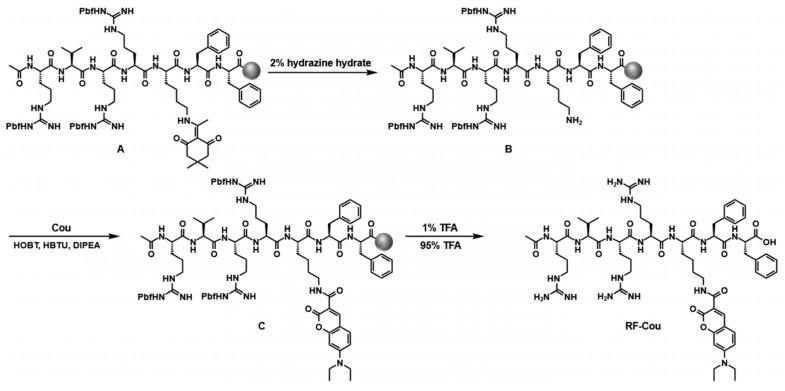
Synthetic route for RF-Cou.

Accordingly, the probe RF-Cou was engineered with the following functional modules: (1) a furin-specific cleavable sequence Arg-Val-Arg-Arg that established the enzymatic recognition site while tuning molecular solubility [34]; (2) a Cou fluorophore linked to the side chain of Lys as the signaling moiety; (3) a well-characterized Phe-Phe moiety to enable molecular self-assembly through π-π stacking and hydrogen bonding after furin-mediated hydrolysis [35]. This design allowed facile preparation of RF-Cou through a single solid-phase synthesis step (Scheme 1). Further characterization through electrospray ionization mass spectrometry (ESI-MS, Figure S1) and nuclear magnetic resonance (NMR, Figures S2 and S3) unambiguously confirmed the structure of RF-Cou. Using an analogous synthetic approach, we obtained the enzymatic cleavage product F-Cou (Scheme S1 and Figures S4–S6) for subsequent investigation.

**Figure 2:**
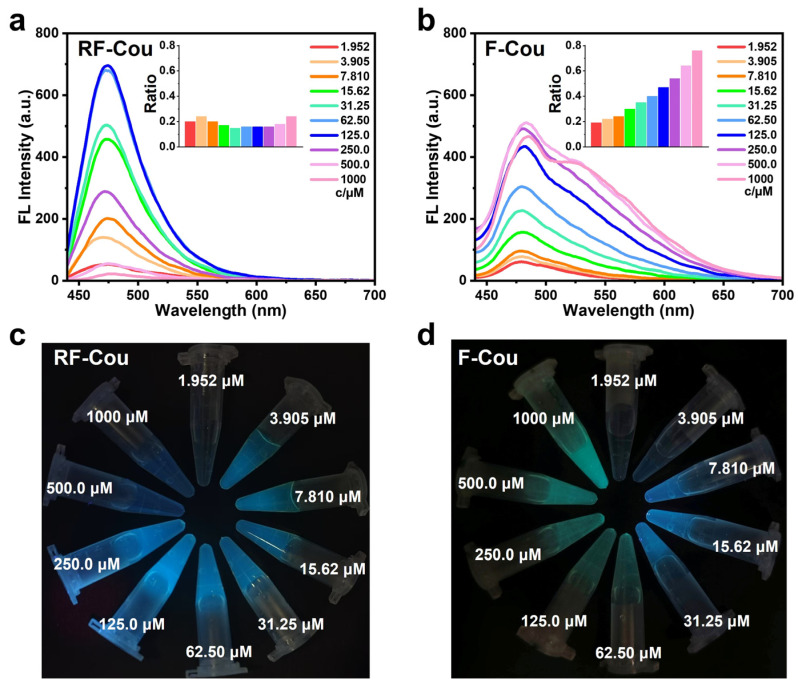
The fluorescence emission spectra and images of RF-Cou (**a**,**c**) and F-Cou (**b**,**d**) at different concentrations in furin working buffer. Inset graphs show the monomer–excimer transition ratios of Cou (calculated by *I*_550_/*I*_475_). The fluorescence spectra were conducted with the excitation of 430 nm and the slit width of 5 nm. The fluorescence images were taken under the 365 nm UV lamp irradiation.

In the next phase, we investigated the optical properties of RF-Cou and its enzymatic product F-Cou. Both RF-Cou and F-Cou exhibited maximum absorption peaks at 430 nm in furin working buffer, which coincided with the characteristic absorption peak of Cou (Figure S7). This observation indicated that the conjugation of the Cou to the peptides did not significantly alter their ultraviolet-visible (UV-Vis) absorption characteristics. However, the fluorescence properties of F-Cou differed significantly from those of RF-Cou. Under UV illumination, RF-Cou exhibited intense blue fluorescence featuring a maximum emission peak at 475 nm, which was attributed to the monomeric form of Cou. The fluorescence intensity initially increased with concentration, peaked at 125 μM, and then declined (Figure 2a,c). Notably, despite pronounced fluorescence quenching at concentrations as high as 1 mM, the emission spectrum of RF-Cou showed no obvious new peaks. In contrast, as the concentration increased, a new emission peak around 550 nm gradually emerged in the fluorescence spectra of F-Cou [15]. When the concentration exceeded 31.25 μM, the solution of F-Cou demonstrated an abrupt color change from blue to green under UV irradiation (Figure 2b,d). These observations confirmed the formation of Cou excimers.

**Figure 3:**
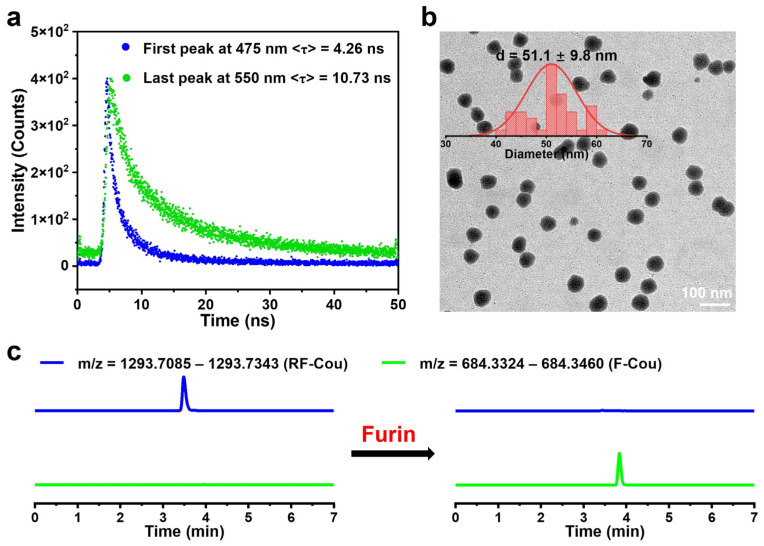
(**a**) Fluorescence decay profiles of the first emission peak at 475 nm (blue) and the last emission peak at 550 nm (green) of 200 μM F-Cou; λ_ex_ = 405 nm. (**b**) TEM image of F-Cou. Inset: the particle size distribution histogram. (**c**) LC-MS traces of 200 μM RF-Cou incubated with or without 50 U/mL furin at 37 °C for 12 h. The blue line corresponds to the probe RF-Cou, and the green line represents its cleavage product F-Cou.

To obtain further proof of the generation of Cou excimers, additional experiments were carried out. Given that the excimers exhibit a longer fluorescence lifetime at their emission peak compared to the monomers, we proceeded to measure the fluorescence lifetimes of the emission peaks in the enzymatic cleavage product F-Cou. As depicted in Figure 3a, the 550 nm emission band of F-Cou displayed a 2.5-fold enhanced fluorescence lifetime relative to the 475 nm band [15], conclusively attributing the 550 nm emission to Cou excimer formation. Next, we examined the factors driving excimer formation. Initially, we evaluated the water solubility of both RF-Cou and its enzymatic product F-Cou through Log *p* value measurement [36]. The data showed that the Log *p* value of RF-Cou was -1.46, while that of Cou was 1.41 (Figure S8), indicating that incorporating the furin-responsive peptide significantly improved the hydrophilicity of Cou. After the hydrophilic furin substrate sequence departed, the remaining F-Cou (Log *p* = 1.15) exhibited increased lipophilicity, facilitating its self-assembly into nanostructures in an aqueous solution. Follow-up experiments demonstrated the formation of nanostructures by the enzymatic cleavage product F-Cou in an aqueous solution. Critical micelle concentration (CMC) testing demonstrated that RF-Cou exhibited poor self-assembly performance in furin working buffer, maintaining >95% transmittance even at elevated concentrations up to 800 μM. Despite this, plots of transmittance of F-Cou versus concentration portrayed two regimes, suggesting that F-Cou could form nanostructures with a CMC of 45.1 μM (Figure S9). Additionally, transmission electron microscopy (TEM) analysis confirmed the self-assembly of F-Cou into monodisperse nanoparticles measuring 51.1 ± 9.8 nm in diameter (Figure 3b), whereas no nanoparticle distribution was observed in the TEM image of RF-Cou (Figure S10), indicating that the enzymatic cleavage product F-Cou possessed the ability for self-assembly. Consistent with these data, dynamic light scattering (DLS) analysis further verified nanoparticle formation in the F-Cou solution, exhibiting a hydrodynamic diameter of 69.2 ± 18.4 nm, while no detectable signal corresponding to nanoparticles was found in the RF-Cou solution (Figure S11). These results indicated that the enzymatic cleavage product of RF-Cou, F-Cou, could spontaneously form nanoparticles in aqueous solution, which was proved to promote the generation of Cou excimers [14,15].

Subsequently, we demonstrated that RF-Cou produced cleavage product F-Cou upon furin-mediated processing in vitro. Specifically, after RF-Cou was maintained in the furin working buffer for 12 h, liquid chromatography–mass spectrometry (LC-MS) analysis detected only the chromatographic peak of RF-Cou (theoretical *m*/*z* = 1293.7085–1293.7343), which eluted at 3.4 min (Figure 3c and Figure S12a). Nevertheless, when RF-Cou was incubated with furin at 37 °C for 12 h, a new peak corresponding to its cleavage product F-Cou (theoretical *m*/*z* = 684.3324–684.3420) appeared in the LC-MS spectra with a retention time of 3.8 min (Figure 3c and Figure S12b), which demonstrated that RF-Cou could be enzymatically processed by furin to generate F-Cou. Based on the above in vitro experiments, we concluded that RF-Cou existed as a blue fluorescent monomer in aqueous solution. Upon the interaction with furin, the resulting product F-Cou could self-assemble into nanoparticles, facilitating the conversion of Cou monomers to excimers and inducing a red shift in emission wavelength. These findings validated the feasibility of using RF-Cou for subsequent precision live-cell imaging studies of furin activity.

**Figure 4:**
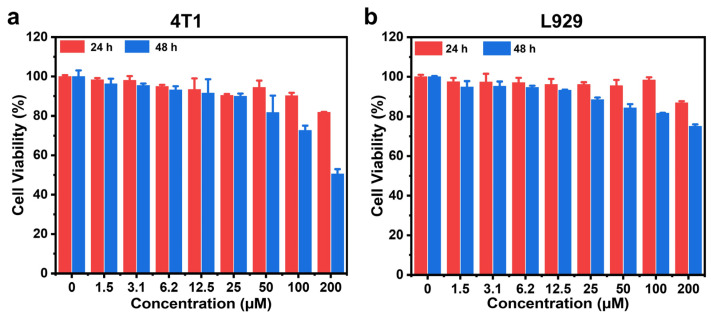
Relative cell viabilities of (**a**) 4T1 tumor cells and (**b**) L929 normal cells after treatment with different concentrations of RF-Cou for 24 h or 48 h, respectively (*n* = 3, mean ± standard deviation (SD)).

Prior to conducting live-cell imaging, we validated the compatibility of RF-Cou within the biological system. UV-Vis absorption spectra of RF-Cou stored in phosphate-buffer saline (PBS), furin working buffer, or cell culture medium within 12 h exhibited no significant alterations, indicating the excellent stability of RF-Cou (Figure S13). Meanwhile, the cytotoxicity of RF-Cou on furin-overexpressing 4T1 mouse breast cancer cells or normal mouse fibroblast L929 cells was evaluated through a 3-(4,5-dimethylthiazol-2-yl)-2,5-diphenyltetrazolium bromide (MTT) assay. After co-incubating RF-Cou with 4T1 or L929 cells for 24 h, no significant cytotoxic effects of RF-Cou were observed across the tested concentration range (0–200 μM) on 4T1 or L929 cells, with cell viability all remaining over 80%. Extended incubation to 48 h revealed minimal toxicity of RF-Cou to L929 cells (cell viability > 80% at 200 μM). However, only about 50% of 4T1 cells survived when the cells were incubated with 200 μM RF-Cou for 48 h (Figure 4). Overall, the cytotoxicity assay advised that RF-Cou had no obvious toxicity to L929 normal cells, but exhibited certain cytotoxicity to furin-overexpressing 4T1 tumor cells in the case of high concentration and long-term exposure. In addition, the hemolysis assay results showed that RF-Cou induced no significant hemolysis in red blood cells (RBCs) at concentrations up to 400 μM, confirming its superior hemocompatibility (Figure S14). The observed compatibility profile in the biological system suggested that RF-Cou was suited for live-cell imaging applications.

**Figure 5:**
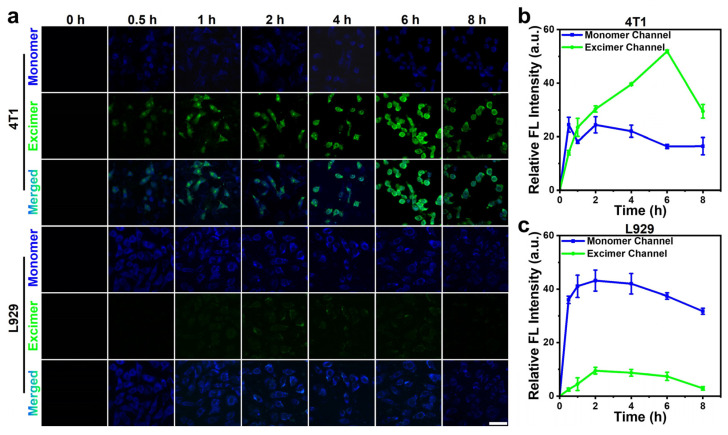
(**a**) Confocal fluorescence images of furin-overexpressing 4T1 cells or normal L929 cells incubated with 200 μM RF-Cou at varying time points; λ_ex_ = 405 nm, monomer channel (blue) λ_em_ = 415–485 nm; excimer channel (green) λ_em_ = 520–600 nm; Scale bar: 50 μm. Time-dependent relative fluorescence intensity in monomer and excimer channels of 4T1 (**b**) or L929 (**c**) cells, respectively (*n* = 3, mean ± SD).

Afterward, we evaluated the imaging capability of RF-Cou for furin activity in cells. Time-course fluorescence imaging of furin-overexpressing 4T1 tumor cells and control L929 normal cells incubated with 200 μM RF-Cou was recorded using a laser scanning confocal microscope. As shown in Figure 5a,b, monomer channel (blue) fluorescence in 4T1 cells exhibited a transient elevation within the initial 0.5 h before stabilizing at a comparatively low level. In the excimer channel (green), the fluorescence intensity progressively increased to a 3.2-fold higher peak at 6 h (vs monomer channel) before decreasing. Nevertheless, in L929 normal cells, both monomer and excimer channel fluorescence intensities increased over time, peaking at 2 h, and then gradually declining. Throughout the observation period, excimer channel fluorescence remained nearly undetectable, while monomer channel fluorescence intensity was markedly higher, which was approximately 5.1-fold greater than excimer channel fluorescence at the time point of 6 h (Figure 5a,c). These phenomena indicated that RF-Cou could be effectively activated preferentially in the Golgi apparatus [37] of furin-overexpressing 4T1 cells, leading to the conversion of Cou from monomer to excimer and thereby “turning on” the excimer’s fluorescent signal.

**Figure 6:**
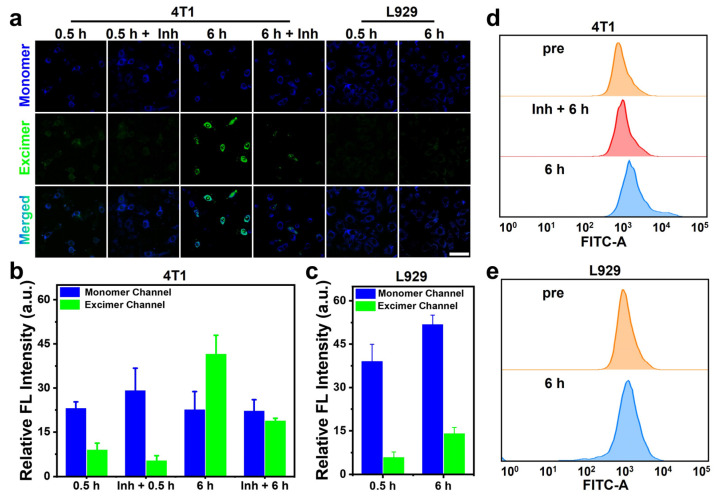
(**a**) Confocal fluorescence images of 4T1 or L929 cells incubated with 200 μM RF-Cou for 0.5 h or 6 h after being treated with or without furin inhibitor (Inh); λ_ex_ = 405 nm, monomer channel (blue) λ_em_ = 415–485 nm; excimer channel (green) λ_em_ = 520–600 nm; Scale bar: 50 μm. Fluorescence intensity in monomer and excimer channels of 4T1 (**b**) or L929 (**c**) cells, respectively (*n* = 3, mean ± SD). Flow cytometry results of the excimer channel fluorescence in 4T1 (**d**) or L929 (**e**) incubated with 200 μM RF-Cou for 6 h after being treated with or without Inh.

To investigate the role of furin in activating excimer fluorescence, we pretreated 4T1 cells with 1 mM of furin inhibitor II (Inh) for 30 min before the initiation of the experiments. After 0.5 h of incubation with RF-Cou, both 4T1 tumor cells (with or without Inh pretreatment) and L929 normal cells displayed moderate monomer fluorescence and relatively weak excimer fluorescence, indicating successful cellular uptake of RF-Cou but inefficient conversion into excimers. Upon extending the incubation time to 6 h, L929 cells presented increased fluorescence in both monomer and excimer channels, though monomer fluorescence was obviously higher than that of excimers. Contrarily, 4T1 cells showed a distinct increase in excimer fluorescence, while monomer fluorescence remained almost unchanged. Notably, when 4T1 cells were pretreated with Inh, a modest increase in excimer fluorescence was observed after 6 h incubation of RF-Cou, which was significantly weaker than that in cells without Inh pretreatment (Figure 6a–c). Flow cytometric analysis further evidenced these results. As depicted in Figure 6d,e, and Figure S15, 4T1 cells incubated with RF-Cou for 6 h demonstrated a pronounced enhancement of fluorescence in the green channel (i.e., excimer channel) compared to the cells before treatment. Meanwhile, Inh-treated 4T1 cells or L929 normal cells showed a faint increase in green fluorescence after 6 h of RF-Cou incubation. These flow cytometry results, along with the fluorescence imaging data, collectively indicated that the excimer fluorescence of RF-Cou was specifically activated in 4T1 tumor cells with elevated furin expression, thereby inducing a characteristic red shift in emission wavelength for furin imaging. All in all, RF-Cou demonstrated specific imaging capability for furin activity, providing a powerful tool for furin imaging in living cells.

## 3. Materials and Methods

### 3.1. Syntheses of RF-Cou

The synthetic route for RF-Cou is shown in Scheme 1. Specifically, solid phase peptide synthesis (SPPS) was used to prepare the peptide Ac-Arg-Val-Arg-Arg-Lys(Dde)-Phe-Phe-OH-Resin (A). Then, to remove the Dde protecting group, compound A was treated with 2% hydrazine in DMF (15 mL) two times, each lasting 5 min, to yield compound B. Subsequently, Cou, 1-hydroxybenzotriazole (HOBt, 2-(1H-benzotriazol-1-yl)-1,1,3,3,-tetramethyluronium hexafluorophosphate (HBTU), and *N, N*-diisopropylethylamine (DIPEA) were added to react with B. After the 4 h reaction, the compound C was prepared. The peptide was cleaved from the resin by 1% trifluoroacetic acid (TFA) in dichloromethane (DCM), and then the Pbf protective group was removed by a deprotection solvent (95% TFA, 3% DCM, and 2% triisopropylsilane (TIPS)) reaction for 3 h to obtain RF-Cou and further through HPLC purification using water–acetonitrile added with 0.1% TFA as the eluent from 7:3 to 1:9. MS: calculated for C_63_H_92_N_18_O_12_ [M + H]^+^: 1293.7214, obsvd. ESI-MS: *m*/*z* 1293.7249 (Figure S1). ^1^H NMR of RF-Cou (400 MHz, DMSO-*d_6_*) δ (ppm): 8.68 (s, 1H), 7.93 (d, *J* = 8.1 Hz, 1H), 7.29–7.21 (m, 10H), 6.83 (dd, *J* = 9.1, 2.4 Hz, 1H), 6.63 (d, *J* = 2.3 Hz, 1H), 4.56–4.51 (m, 1H), 4.48–4.43 (m, 1H), 4.29–4.20 (m, 5H), 3.50 (q, *J* = 7.0 Hz, 4H), 3.30–2.66 (m, 12H), 1.96 (td, *J* = 12.1, 10.9, 5.4 Hz, 1H), 1.88 (s, 3H), 1.79–1.31 (m, 16H), 1.31–1.19 (m, 2H), 1.15 (t, *J* = 7.0 Hz, 6H), 0.83 (dd, *J* = 13.3, 6.7 Hz, 6H) (Figure S2). ^13^C NMR of RF-Cou (100 MHz, DMSO-*d*_6_) δ (ppm): 173.13 (1C), 172.11 (1C), 171.70 (1C), 171.59 (1C), 171.31 (1C), 171.02 (1C), 170.56 (1C), 162.66 (1C), 162.30 (1C), 159.08 (1C), 157.68 (1C), 157.24 (1C), 157.21 (1C), 152.91 (1C), 148.14 (1C), 137.87 (1C), 137.78 (1C), 132.06 (1C), 129.61 (2C), 129.57 (2C), 128.65 (2C), 128.42 (2C), 126.92 (1C), 126.67 (1C), 118.79 (1C), 115.84 (1C), 110.64 (1C), 108.12 (1C), 96.30 (1C), 57.88 (1C), 53.97 (1C), 53.89 (1C), 52.84 (1C), 52.78 (1C), 52.68 (1C), 52.65 (1C), 52.45 (1C), 44.82 (2C), 40.91 (3C), 38.03 (1C) 37.19 (1C), 32.38 (1C), 31.13 (1C), 29.63 (1C), 29.39 (3C), 25.57 (1C), 25.48 (1C), 25.42 (1C), 23.30 (1C), 22.91 (1C), 19.64 (1C), 18.32 (1C), 12.78 (2C) (Figure S3).

### 3.2. Syntheses of F-Cou

The synthetic route for F-Cou is shown in Scheme S1. Briefly, SPPS was used to prepare the peptide Boc-Lys(Fmoc)-Phe-Phe-OH-Resin (D). Then, the Fmoc protecting group of compound D was treated with 20% piperidine in DMF (20 mL) for 30 min to obtain compound E. After Cou was linked to the side chain of Lys, the compound was cleaved and deprotected to produce F-Cou and further through HPLC purification using water–acetonitrile added with 0.1% TFA as the eluent from 50:50 to 5:95. MS: calculated for C_38_H_45_N_5_O_7_ (M + H)^+^: 684.3391, obsvd. ESI-MS (M + H)^+^: *m*/*z* 684.3395 (Figure S4). ^1^H NMR of F-Cou (400 MHz, DMSO–*d_6_*) δ (ppm): 8.66 (s, 1H), 7.68 (d, *J* = 9.0 Hz, 1H), 7.28–7.20 (m, 10H), 6.81 (dd, *J* = 9.1, 2.4 Hz, 1H), 6.61 (d, *J* = 2.3 Hz, 1H), 4.62 (td, *J* = 8.9, 4.5 Hz, 1H), 4.49 (td, *J* = 8.3, 5.2 Hz, 1H), 3.48 (q, *J* = 7.0 Hz, 4H), 3.26 (m, *J* = 6.5 Hz, 2H), 3.05 (ddd, *J* = 30.9, 14.0, 4.9 Hz, 2H), 2.96–2.87 (m, 2H), 2.83–2.73 (m, 1H), 1.68 (td, *J* = 13.8, 7.7 Hz, 2H), 1.49 (p, *J* = 7.8 Hz, 2H), 1.31 (p, *J* = 8.0 Hz, 2H), 1.14 (t, *J* = 7.0 Hz, 6H) (Figure S5). ^13^C NMR of F-Cou (100 MHz, DMSO-d6) δ (ppm): 172.57 (1C), 170.59 (1C), 168.53 (1C), 162.11 (1C), 161.75 (1C), 157.18 (1C), 152.41 (1C), 147.61 (1C), 137.43 (1C), 137.35 (1C), 131.52 (1C), 129.16 (2C), 129.06 (2C), 128.16 (2C), 128.12 (2C), 126.36 (2C), 110.13 (1C), 109.42 (1C), 107.63 (1C), 95.84 (1C), 54.00 (1C), 53.36 (1C), 51.98 (1C), 44.31 (2C), 38.69 (1C), 37.48 (1C), 36.68 (1C), 31.05 (1C), 28.79 (1C), 21.70 (1C), 12.29 (2C) (Figure S6).

### 3.3. Critical Micelle Concentration (CMC) Measurement

A series of RF-Cou and F-Cou solutions at different concentrations were prepared in the furin working buffer. The transmittance spectra of the solutions were measured using a UV-3600 spectrophotometer (SHIMADZU, Kyoto, Japan).

### 3.4. Stability Measurement

To test the chemical stability, RF-Cou (50 μM) was incubated in PBS, furin working buffer (20 mM 4-(2-hydroxyerhyl) piperazine-1-erhanesulfonic acid (HEPES), 1 mM CaCl_2_, and 0.2 mM β-mercaptoethanol, pH = 7.5), and cell culture medium (1640 medium containing 10% fetal bovine serum (FBS)). Then, the absorption spectra of RF-Cou were recorded with a microplate reader at varying time points (0, 3, 6, and 12 h).

### 3.5. Cell Culture

4T1 mouse breast cancer cells or L929 mouse fibroblast cells were cultured in RPMI-1640 medium or Dulbecco’s Modified Eagle’s medium (DMEM) supplemented with 10% *v*/*v* FBS, 100 U/mL penicillin, 100 μg/mL streptomycin, and 0.25 μg/mL amphotericin B, respectively. These cells were incubated at 37 °C in a humidified atmosphere of 5% CO_2_.

### 3.6. Cytotoxicity Assay

First, the cells (4T1, L929 cell lines) were seeded in 96-well plates (3 × 10^3^ cells in each well) and incubated in a 37 °C cell incubator for 24 h. The RPMI-1640 medium or DMEM was replaced with different concentrations of RF-Cou (0, 1.5, 3.1, 6.2, 12.5, 25.0, 50.0, 100, and 200 μM) in the medium and incubated for 24 h or 48 h, respectively. MTT (5 mg/mL, 10 μL) was then added to each well, and the cells were incubated for another 4 h. The formed formazan crystals were dissolved by DMSO (100 μL per well), and the absorbance at 570 nm was measured using a microplate reader (BioTek, Winooski, VT, USA).

### 3.7. Hemolysis Assay

A total of 10 μL RBC solution was added to 0.5 mL RF-Cou (dissolved in PBS) at different concentrations to obtain a final RF-Cou concentration of 25, 50, 100, 200, and 400 μM, respectively. RBCs dispersed separately in PBS were set as a negative control, while double distilled water (ddwater) was set as a positive control. The mixture was incubated at 37 °C for 1 h and then centrifuged at 3000 rpm for 10 min. After pictures were taken, the supernatant (100 μL) was placed in 96-well plates, and the absorbance of the released hemoglobin at 540 nm in each group was measured using a microplate reader. The hemolysis rate was calculated by using the following equation:

Hemolysis Rate (%) = (OD_Sample_ − OD_PBS_)/(OD_ddWater_ − OD_PBS_) × 100%.

### 3.8. Cell Imaging

4T1 cells or L929 cells were seeded on confocal dishes (1 × 10^5^ cells/dish) and incubated at 37 °C in a humidified atmosphere of 5% CO_2_ for 24 h. Following the removal of the conditioned medium, 4T1 cells or L929 cells were treated with RF-Cou (200 μM) at various times (0, 0.5, 1, 2, 4, 6, and 8 h). After the incubation was completed, the medium was discarded, PBS was added to clean the cells three times, and laser confocal scanning microscopy was used for cell imaging. To investigate the role of furin in activating excimer fluorescence, 4T1 cells were pre-incubated with 1 mM of furin inhibitor II (Inh) for 30 min and then treated with RF-Cou (200 μM) for 0.5 h or 6 h before imaging.

## 4. Conclusions

In summary, we developed a furin-triggered peptide self-assembling fluorescent probe RF-Cou for precision live-cell imaging. RF-Cou showed monomer fluorescence, emitting at 475 nm. Under the specific hydrolysis of furin, RF-Cou generated an amphiphilic cleavage product F-Cou, which could self-assemble into nanoparticles and promote the generation of Cou excimers to induce a red shift in the fluorescence emission to 550 nm. Crucially, RF-Cou selectively activated excimer fluorescence in furin-overexpressing 4T1 tumor cells, whereas no significant responsive signal was detected in L929 normal cells or furin-inhibited 4T1 tumor cells, demonstrating its specificity toward furin. We anticipate that this work will provide a highly specific imaging tool for detecting the activity of furin in living cells, demonstrating the considerable promise of peptide-based small molecular probes for biomedical applications.

## Data Availability

Data are contained within the article and Supplementary Materials.

## Supplementary Materials

The following supporting information can be downloaded https://www.mdpi.com/article/10.3390/molecules30112465/s1: Experimental materials and instruments. Figures S1–S3. ESI-MS spectrum, ^1^H NMR and ^13^C NMR spectra of RF-Cou; Scheme S1. The synthesis route to F-Cou; Figure S4–S6. ESI-MS spectrum, ^1^H NMR and ^13^C NMR spectra of F-Cou; Figure S7. UV–Vis spectra of RF-Cou, F-Cou, and Cou; Figure S8. Log P values for RF-Cou, F-Cou, and Cou; Figure S9. CMC values of RF-Cou and F-Cou; Figure S10. TEM image of RF-Cou; Figure S11. Particle size distribution histogram of F-Cou and RF-Cou; Figure S12. Mass spectrometric results in the LC-MS chromatogram of RF-Cou with/without furin incubation in Figure 3a; Figure S13. Absorption spectra of RF-Cou in PBS, furin working buffer and 1640 medium containing 10% FBS; Figure S14. Hemolysis results of RF-Cou; Figure S15. Corresponding mean fluorescence intensity derived from flow cytometric analysis in Figure 6d,e.

## References

[1] Carr J.A., Franke D., Caram J.R., Perkinson C.F., Saif M., Askoxylakis V., Datta M., Fukumura D., Jain R.K., Bawendi M.G. (2018). Shortwave Infrared Fluorescence Imaging with the Clinically Approved Near-Infrared Dye Indocyanine Green. Proc. Natl. Acad. Sci. USA.

[2] Zhang X., Li S., Ma H., Wang H., Zhang R., Zhang X.D. (2022). Activatable NIR-II Organic Fluorescent Probes for Bioimaging. Theranostics.

[3] Wu X., Wang R., Kwon N., Ma H., Yoon J. (2022). Activatable Fluorescent Probes for in Situ Imaging of Enzymes. Chem. Soc. Rev..

[4] Ding L., Xu F., Luo B., Cheng L., Huang L., Jia Y., Ding J. (2024). Preparation of Hematoporphyrin-Poly(Lactic Acid) Nanoparticles Encapsulated Perfluoropentane/Salicylic Acid for Enhanced US/CEST MR Bimodal Imaging. Int. J. Nanomed..

[5] Haider A., Deng X., Mastromihalis O., Pfister S.K., Jeppesen T.E., Xiao Z., Pham V., Sun S., Rong J., Zhao C. (2023). Structure-Activity Relationship of Pyrazol-4-yl-pyridine Derivatives and Identification of A Radiofluorinated Probe for Imaging the Muscarinic Acetylcholine Receptor M_4_. Acta Pharm. Sin. B.

[6] Zhang M., Guan Y., Dang Z., Zhang P., Zheng Z., Chen L., Kuang W., Wang C., Liang G. (2020). Directly Observing Intracellular Nanoparticle Formation with Nanocomputed Tomography. Sci. Adv..

[7] Zeng Z., Liew S.S., Wei X., Pu K. (2021). Hemicyanine-Based Near-Infrared Activatable Probes for Imaging and Diagnosis of Diseases. Angew. Chem. Int. Ed..

[8] Guo R.Y., Wang H.M., Dong X., Hu Y., Li J., Zang Y., Li X. (2021). Selectivity Comparison of Tumor-Imaging Probes Designed Based on Various Tumor-Targeting Strategies: A Proof of Concept Study. ACS Appl. Bio Mater..

[9] Duan Q.J., Zhao Z.Y., Zhang Y.J., Fu L., Yuan Y.Y., Du J.Z., Wang J. (2023). Activatable Fluorescent Probes for Real-Time Imaging-Guided Tumor Therapy. Adv. Drug Deliv. Rev..

[10] Xu L., Liu N., Zhan W., Deng Y., Chen Z., Liu X., Gao G., Chen Q., Liu Z., Liang G. (2022). Granzyme B Turns Nanoparticle Fluorescence “On” for Imaging Cytotoxic T Lymphocyte Activity in Vivo. ACS Nano.

[11] Fujita K., Urano Y. (2024). Activity-Based Fluorescence Diagnostics for Cancer. Chem. Rev..

[12] Chen Y. (2022). Recent Advances in Excimer-Based Fluorescence Probes for Biological Applications. Molecules.

[13] Dixit S.J.N., Wadawale A.P., Ghosh R., Agarwal N. (2024). Excited State Dynamics of Bay and Peri Benzothienyl Perylene to Understand the Excimer Formation and its Dissociation. J. Photochem. Photobiol. A-Chem..

[14] Zhan J., Huang J., Xiao Q., Yu Z.A., Wang Y., Wang X., Liu F., Cai Y., Yang Z., Zheng L. (2024). Optimized Two-Photon Imaging by Stimuli-Responsive Peptide Self-Assembly Facilitates Self-Assisted Counteraction of Cisplatin-Resistance in Cancer Cells. Anal. Chem..

[15] Gao G., Sun X., Liu X., Tang R., Wang M., Zhan W., Zheng J., Liang G. (2022). FAP-α-Instructed Coumarin Excimer Formation for High Contrast Fluorescence Imaging of Tumor. Nano Lett..

[16] Zhang Y., Zhang G., Zeng Z., Pu K. (2022). Activatable Molecular Probes for Fluorescence-Guided Surgery, Endoscopy and Tissue Biopsy. Chem. Soc. Rev..

[17] Ling C.C., Sun T., Chen F., Wu H., Tao W., Xie X., Ji D., Gao G., Chen J., Ling Y. (2023). Precise Tumor Delineation in Clinical Tissues Using a Novel Acidic Tumor Microenvironment Activatable Near-Infrared Fluorescent Contrast Agent. Anal. Chim. Acta.

[18] Klockow J.L., Hettie K.S., LaGory E.L., Moon E.J., Giaccia A.J., Graves E.E., Chin F.T. (2020). An Activatable NIR Fluorescent Rosol for Selectively Imaging Nitroreductase Activity. Sens. Actuators B Chem..

[19] Wang C., Du W., Zhang T., Liang G. (2020). A Bioluminescent Probe for Simultaneously Imaging Esterase and Histone Deacetylase Activity in a Tumor. Anal. Chem..

[20] He Z., Khatib A.M., Creemers J.W.M. (2022). The Proprotein Convertase Furin in Cancer: More than an Oncogene. Oncogene.

[21] Chen P., Wang H., Wu H., Zou P., Wang C., Liu X., Pan Y., Liu Y., Liang G. (2021). Intracellular Synthesis of Hybrid Gallium-68 Nanoparticle Enhances MicroPET Tumor Imaging. Anal. Chem..

[22] Yuan Y., Zhang J., Qi X., Li S., Liu G., Siddhanta S., Barman I., Song X., McMahon M.T., Bulte J.W.M. (2019). Furin-Mediated Intracellular Self-Assembly of Olsalazine Nanoparticles for Enhanced Magnetic Resonance Imaging and Tumour Therapy. Nat. Mater..

[23] Siegfried G., Descarpentrie J., Evrard S., Khatib A.M. (2020). Proprotein Convertases: Key Players in Inflammation-Related Malignancies and Metastasis. Cancer Lett..

[24] Jaaks P., Bernasconi M. (2017). The Proprotein Convertase Furin in Tumour Progression. Int. J. Cancer.

[25] Chen J., Zou X. (2019). Self-Assemble Peptide Biomaterials and Their Biomedical Applications. Bioact. Mater..

[26] Song Z., Han Z., Lv S., Chen C., Chen L., Yin L., Cheng J. (2017). Synthetic Polypeptides: From Polymer Design to Supramolecular Assembly and Biomedical Application. Chem. Soc. Rev..

[27] Deng C., Wu J.T., Cheng R., Meng F.H., Klok H.A., Zhong Z.Y. (2014). Functional Polypeptide and Hybrid Materials: Precision Synthesis Via α-Amino Acid N-Carboxyanhydride Polymerization and Emerging Biomedical Applications. Prog. Polym. Sci..

[28] Gao J., Zhan J., Yang Z. (2020). Enzyme-Instructed Self-Assembly (EISA) and Hydrogelation of Peptides. Adv. Mater..

[29] Zhou J., Xu B. (2015). Enzyme-Instructed Self-Assembly: A Multistep Process for Potential Cancer Therapy. Bioconjugate Chem..

[30] Wang Y., Weng J., Wen X., Hu Y., Ye D. (2021). Recent Advances in Stimuli-Responsive in Situ Self-Assembly of Small Molecule Probes for in Vivo Imaging of Enzymatic Activity. Biomater. Sci..

[31] Liu H.W., Chen L., Xu C., Li Z., Zhang H., Zhang X.B., Tan W. (2018). Recent Progresses in Small-Molecule Enzymatic Fluorescent Probes for Cancer Imaging. Chem. Soc. Rev..

[32] Zhong Y., Zhan J., Xu G., Chen Y., Qin Q., Liao X., Ma S., Yang Z., Cai Y. (2021). Enzyme-Instructed Self-Assembly Enabled Monomer-Excimer Transition to Construct Higher Ordered Luminescent Supramolecular Assembly for Activity-Based Bioimaging. Angew. Chem. Int. Ed..

[33] Kim B.J., Xu B. (2020). Enzyme-Instructed Self-Assembly for Cancer Therapy and Imaging. Bioconjugate Chem..

[34] Thomas G. (2002). Furin at the Cutting Edge: From Protein Traffic to Embryogenesis and Disease. Nat. Rev. Mol. Cell Biol..

[35] Yoo H., Yang J., Yousef A., Wasielewski M.R., Kim D. (2010). Excimer Formation Dynamics of Intramolecular π-Stacked Perylenediimides Probed by Single-Molecule Fluorescence Spectroscopy. J. Am. Chem. Soc..

[36] Jesus A.R., Soromenho M.R.C., Raposo L.R., Esperança J., Baptista P.V., Fernandes A.R., Reis P.M. (2019). Enhancement of Water Solubility of Poorly Water-Soluble Drugs by New Biocompatible N-Acetyl Amino Acid N-Alkyl Cholinium-Based Ionic Liquids. Eur. J. Pharm. Biopharm..

[37] Li X., Cao C., Wei P., Xu M., Liu Z., Liu L., Zhong Y., Li R., Zhou Y., Yi T. (2019). Self-Assembly of Amphiphilic Peptides for Recognizing High Furin-Expressing Cancer Cells. ACS Appl. Mater. Interfaces.

