# Supplementary material for: Furin-Triggered Peptide Self-Assembly Activates Coumarin Excimer Fluorescence for Precision Live-Cell Imaging
Source: Molecules. 2025 Jun 4;30(11):2465. doi: 10.3390/molecules30112465 (PMC12156344; doi:10.3390/molecules30112465)
Supplement: Supplementary file 1 [file molecules-30-02465-s001.zip › molecules-3663726-supplementary.pdf]

Supplementary Materials

Furin-Triggered Peptide Self-Assembly Activates Coumarin Excimer Fluorescence for Precision Live-Cell Imaging

Peiyao Chen[1], Liling Meng[1], Yuting Wang[1], Xiaoya Yan[1], Meiqin Li[2], Yun Deng[3] and Yao Sun[2*]

1. Key Laboratory of Fermentation Engineering (Ministry of Education), National "111" Center for Cellular Regulation and Molecular Pharmaceutics, Hubei Key Laboratory of Industrial Microbiology, School of Life and Health Sciences, Hubei University of Technology, Wuhan 430068, China

2. National Key Laboratory of Green Pesticide, College of Chemistry, Central China Normal University, Wuhan 430079, China

3. Key Laboratory of Flexible Optoelectronic Materials and Technology, Ministry of Education, Jianghan University, Wuhan 430056, China

*Correspondence: sunyaogbasp@ccnu.edu.cn

Contents:

1. Experimental Materials and Instruments

2. Supporting Figures

1. Experimental Materials and Instruments

All starting materials and reagents were purchased from commercial suppliers and applied without further purification. 2-Cl-trityl chloride resin and Fmoc-amino acids were obtained from GL Biochem (Shanghai, China). 3-(4,5-Dimethylthiazol-2-yl)-2,5-diphenyltetrazolium bromide (MTT) was purchased from Beyotime Biotechnology. Furin (2000 U/mL) was supplied by NEB (P8077S). All peptides were purified using a Wufeng LC-100 high-performance liquid chromatography (HPLC) system, which was equipped with an LC-UV100 ultraviolet detector and dual LC-P100 high-pressure constant-flow pumps. The mobile phase consisted of CH_3CN (0.1% of trifluoroacetic acid (TFA)) and water (0.1% of TFA). For NMR analysis, ^{1}H NMR and ^{13}C NMR spectra were recorded on Varian Unity Inova 400. High-resolution liquid chromatography-mass spectrometry (LC-MS) spectrums were obtained from a high-resolution liquid chromatography-mass spectrometry (ThermoFisher Corporation, USA). UV-Vis absorption spectra were measured with a UV-3600 spectrophotometer (SHIMADZU, Japan). Fluorescence spectra were recorded by an LS55 fluorescence spectrometer (Perkin Elmer, USA). Optical density was measured using a microplate reader (BioTek, USA). The size distribution of nanoparticles was determined by DLS using a Malvern Zetasizer Nano ZS+MPT-2 nanoparticle potentiometer (Malvern, Germany). The decay lifetimes of fluorescence were assessed using a steady-state/transient-state fluorescence spectrometer (Edinburgh Instruments, U.K.). TEM images were obtained with a JEM-1400Plus transmission electron microscope. *In vitro* fluorescent images were acquired with a laser scanning confocal microscope (Leica TCS SP8, Germany). Quantitative analysis of intracellular fluorescence was carried out via flow cytometry (Beckman, USA).

2. Supporting Figures

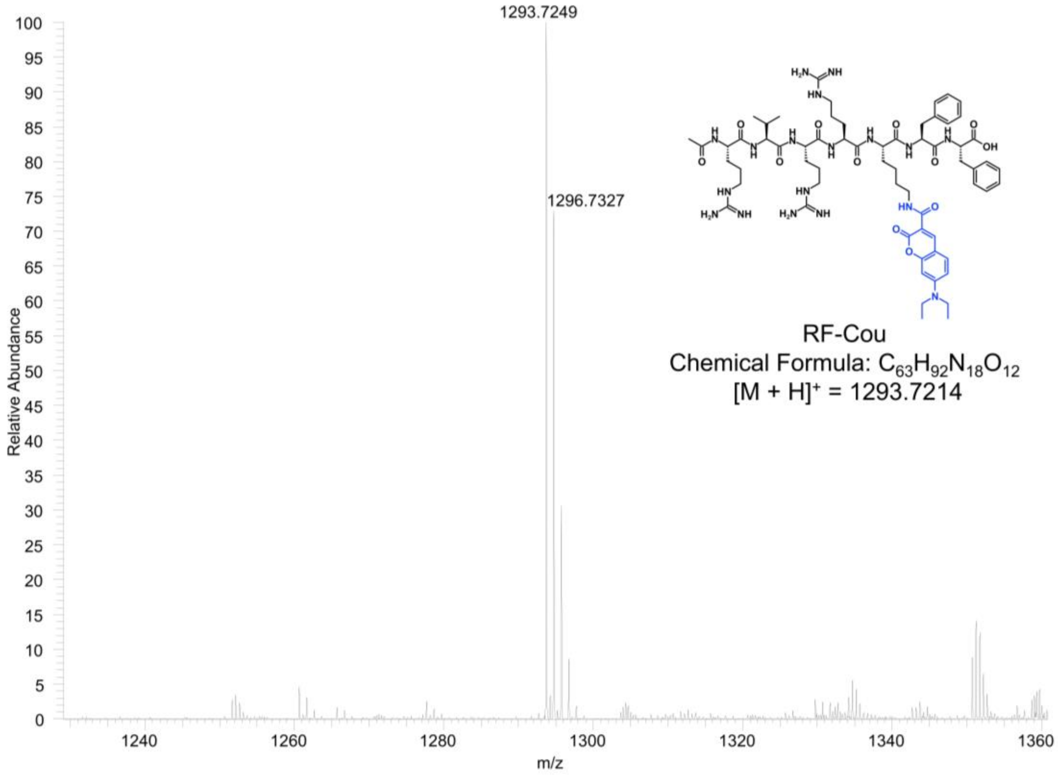

Figure S1. ESI-MS spectrum of RF-Cou.

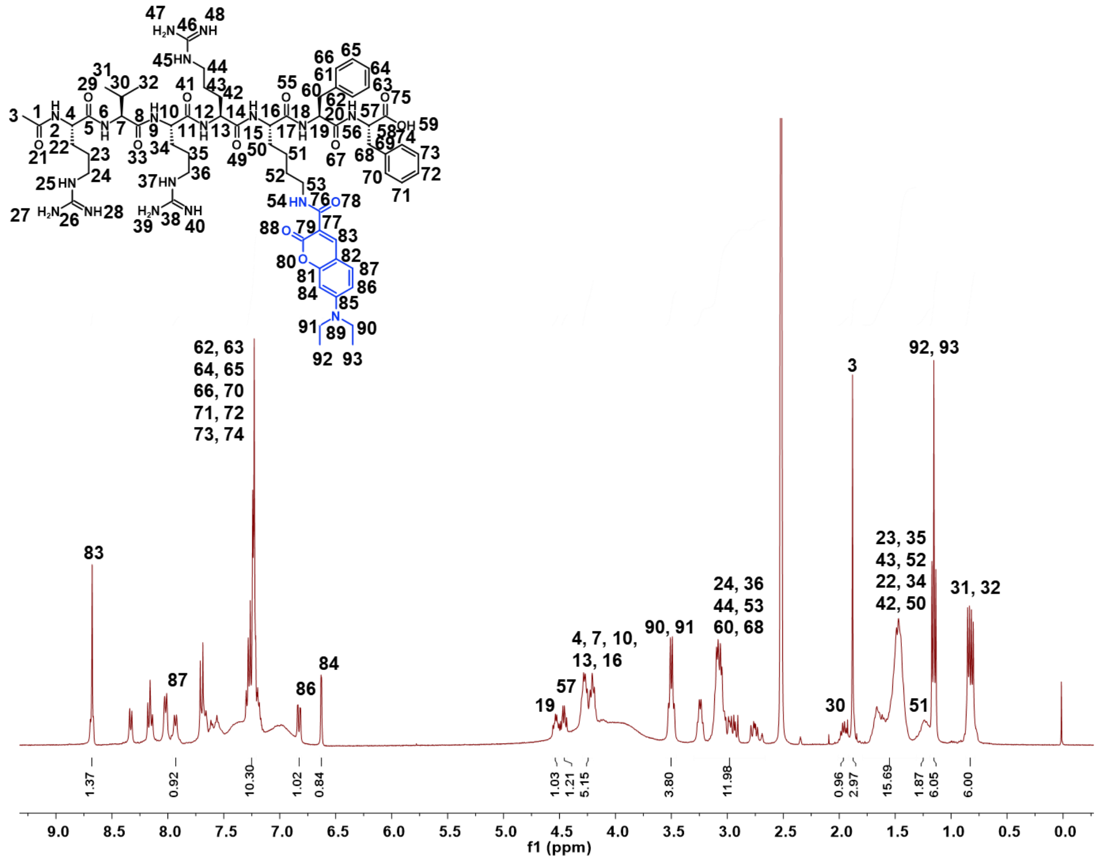

Figure S2. ^1H NMR spectrum of RF-Cou in DMSO-d_6.

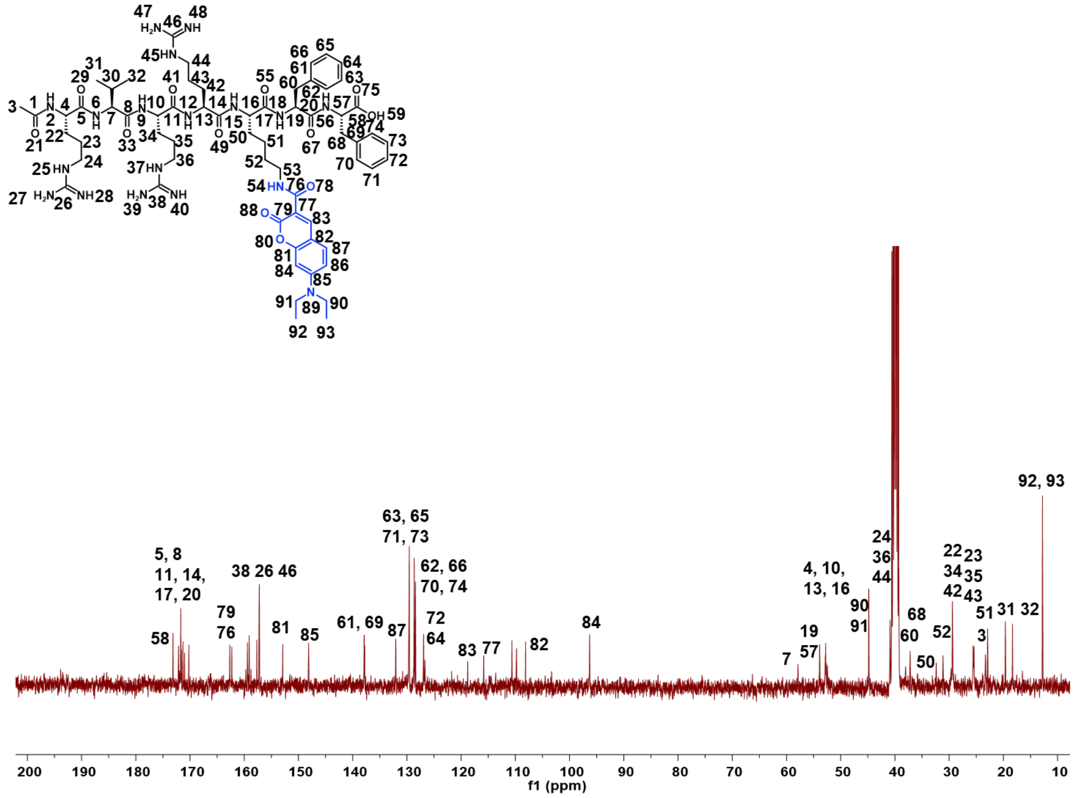

Figure S3. ^{13}C NMR spectrum of RF-Cou in DMSO-d_6.

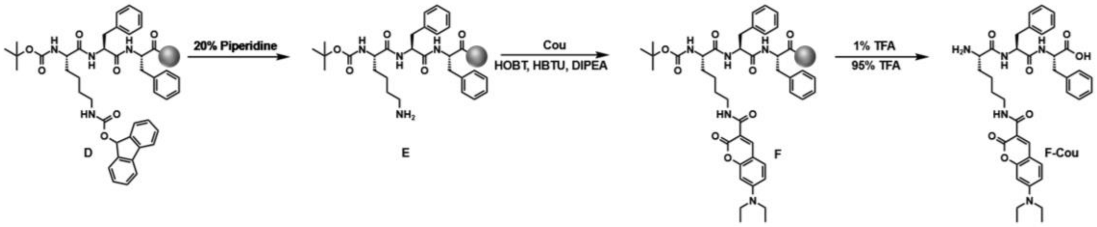

Scheme S1. Synthetic route for F-Cou.

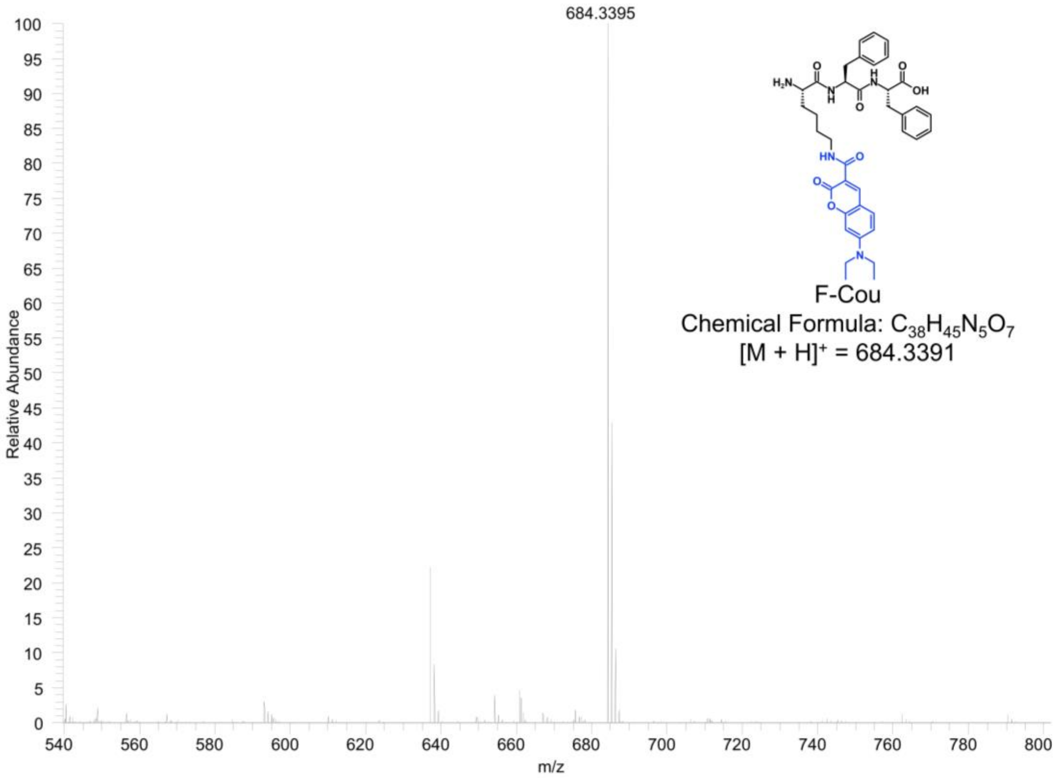

Figure S4. Mass spectrum of F-Cou.

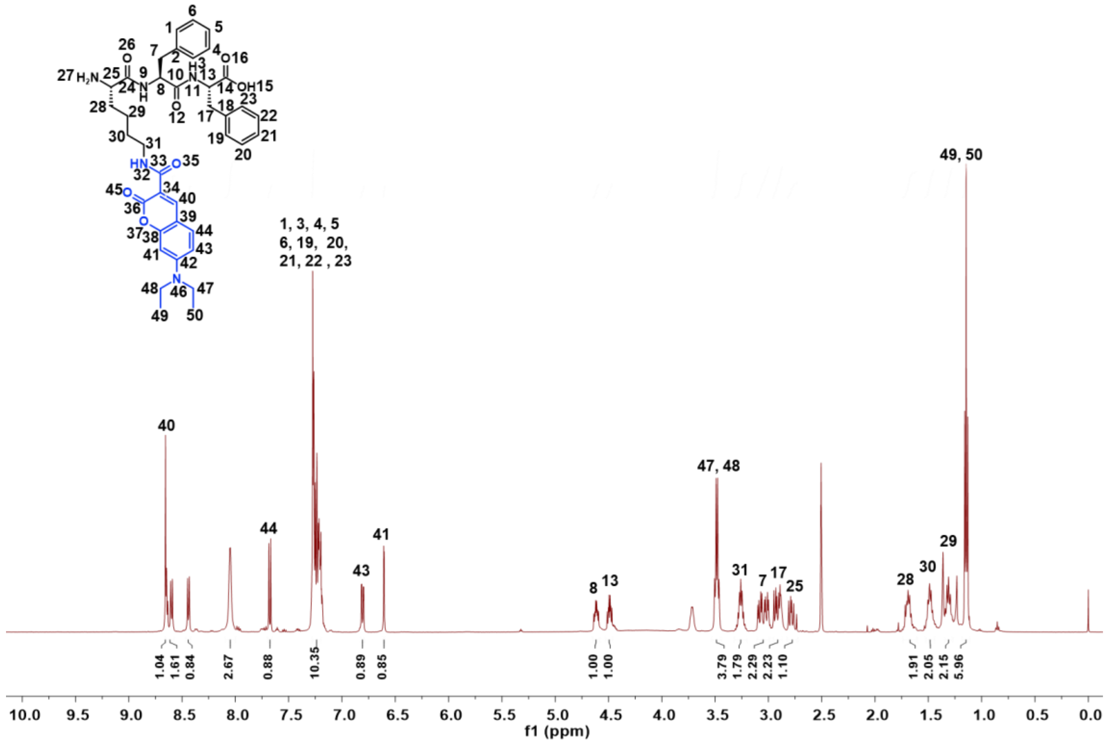

Figure S5. ^{1}H NMR spectrum of F-Cou in DMSO-d_6.

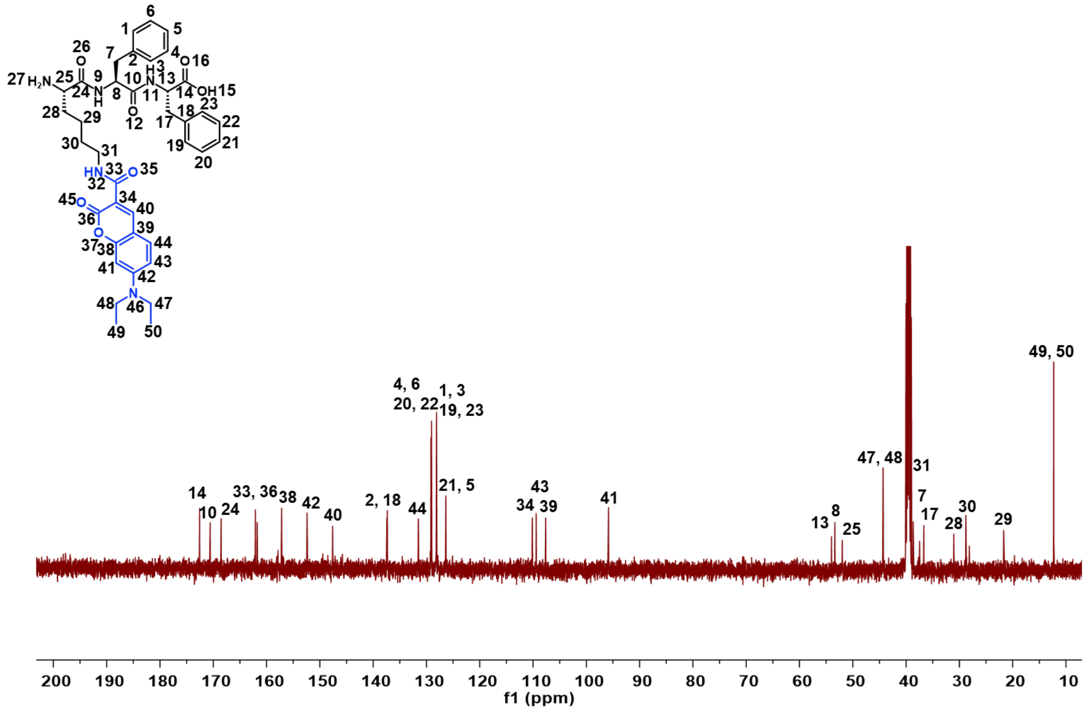

Figure S6. ^{13}C NMR spectrum of F-Cou in DMSO-d_6.

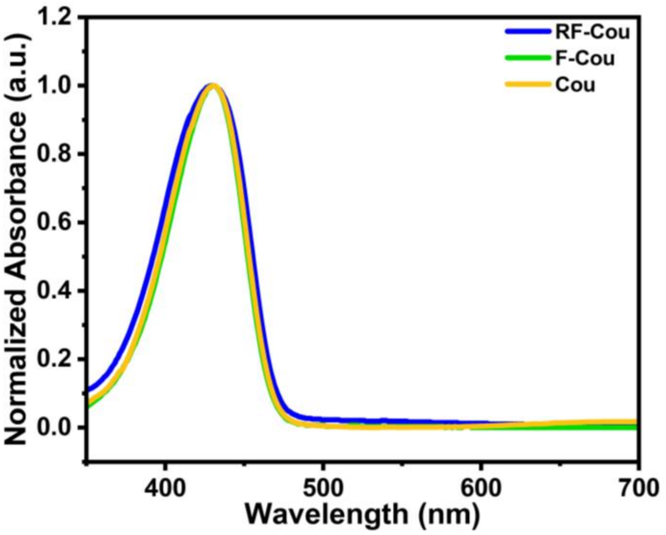

Figure S7. UV-Vis absorption spectra of RF-Cou, F-Cou, and Cou in furin working buffer.

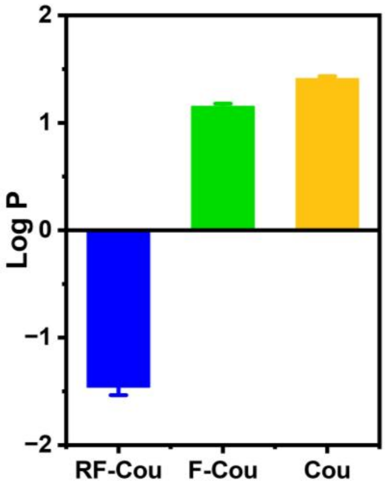

Figure S8. Log P values for RF-Cou, F-Cou, and Cou (n = 3, mean ± SD).

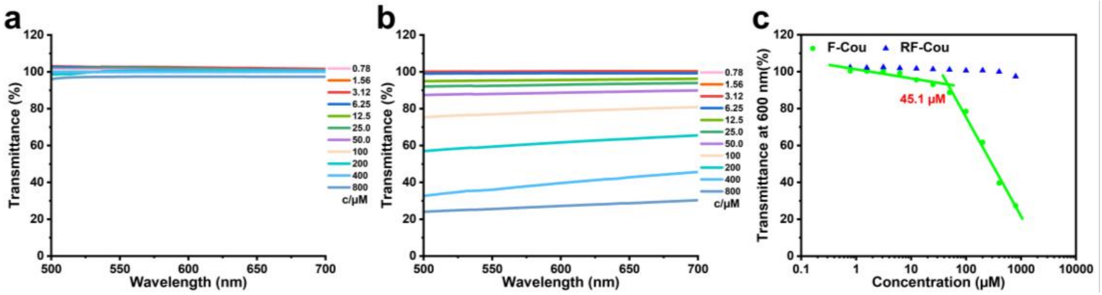

Figure S9. Transmittance spectra of RF-Cou (a) or F-Cou (b) at different concentrations. (c) Plotted transmittance-concentration curve of RF-Cou and F-Cou at 600 nm for the determination of CMC.

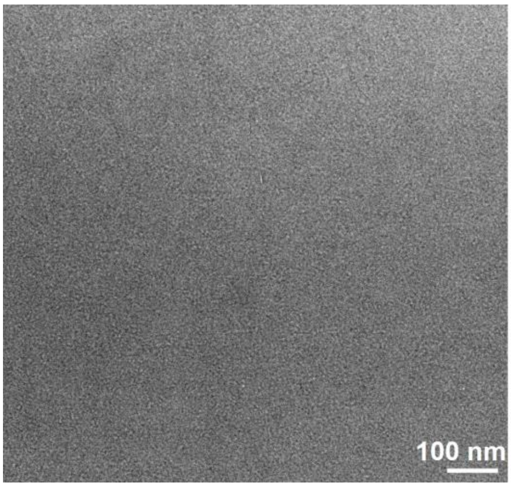

Figure S10. TEM image of RF-Cou.

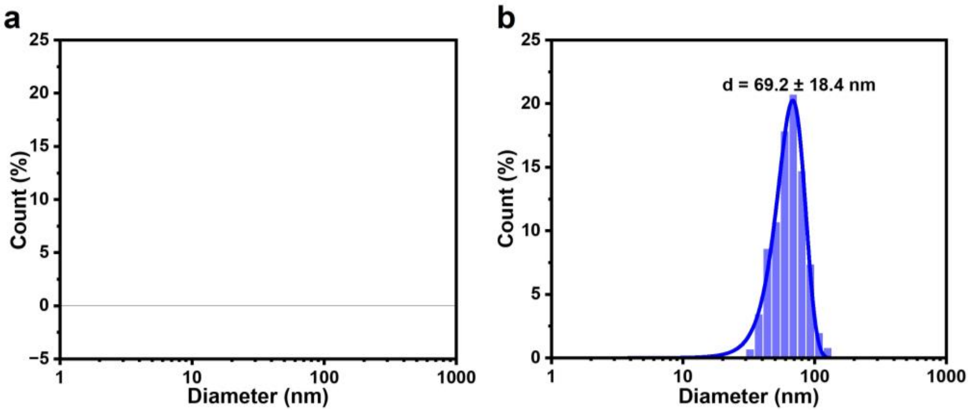

Figure S11. The particle size distribution histogram of RF-Cou (a) or F-Cou (b).

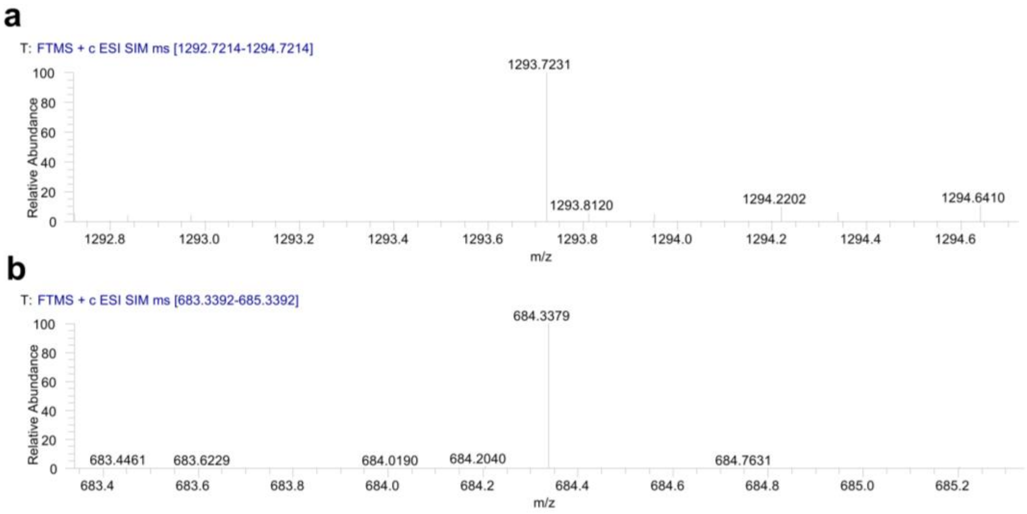

Figure S12. (a) Mass spectrometric result of the 3.4 min chromatographic peak (blue line) in the LC-MS chromatogram of RF-Cou without furin incubation in Figure 3c. (b) Mass spectrometric result of the 3.8 min chromatographic peak (green line) in the LC-MS chromatogram of RF-Cou with furin incubation in Figure 3c.

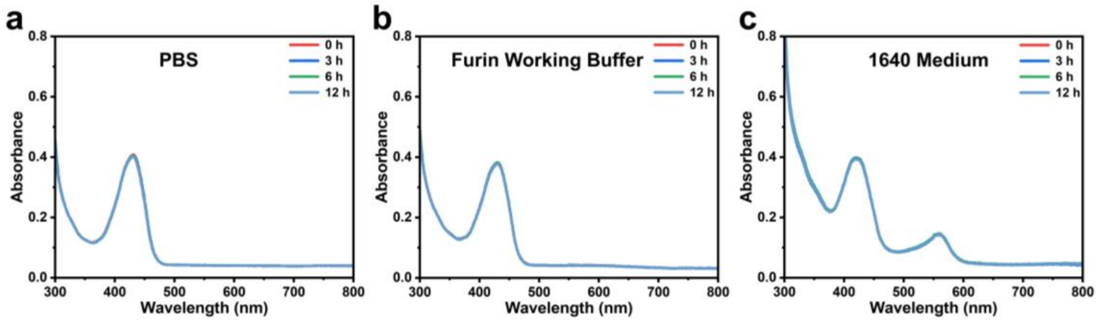

Figure S13. UV-Vis absorption spectra of RF-Cou in (a) PBS, (b) furin working buffer, and (c) 1640 medium containing 10 % FBS recorded at different times.

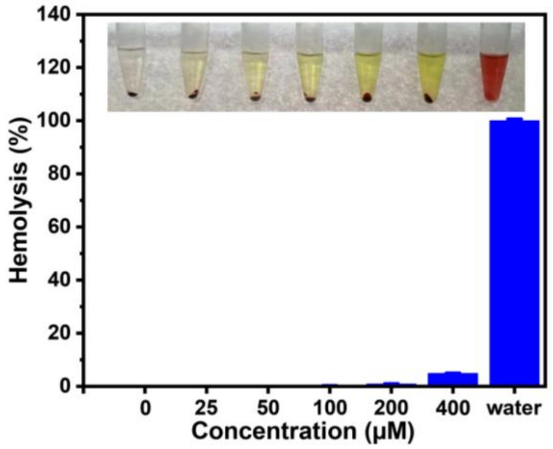

Figure S14. Hemolysis results of RF-Cou at various concentrations. RBCs in PBS and water were set as the negative and positive control, respectively (n = 3, mean ± SD).

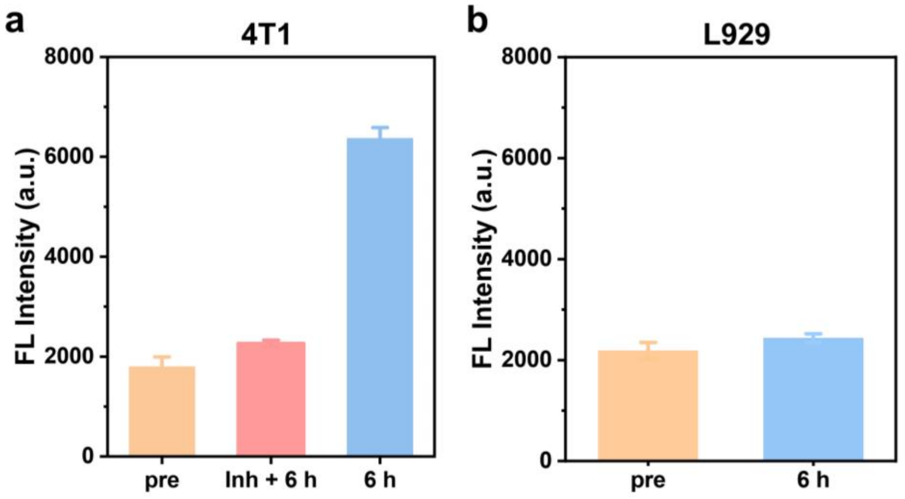

Figure S15. The corresponding mean fluorescence intensity derived from flow cytometric analysis in Figure 6d (a) and e (b) (n = 3, mean ± SD).
